# MRI cT1–2 rectal cancer staging accuracy: a population‐based study

**DOI:** 10.1002/bjs.11590

**Published:** 2020-04-16

**Authors:** R. Detering, S. E. van Oostendorp, V. M. Meyer, S. van Dieren, A. C. R. K. Bos, J. W. T. Dekker, O. Reerink, J. H. T. M. van Waesberghe, C. A. M. Marijnen, L. M. G. Moons, R. G. H. Beets‐Tan, R. Hompes, H. L. van Westreenen, P. J. Tanis, J. B. Tuynman, Dutch ColoRectal Audit Group*

**Affiliations:** ^1^ Department of Surgery, Cancer Centre Amsterdam, Amsterdam UMC University of Amsterdam Amsterdam the Netherlands; ^2^ Clinical Research Unit, Amsterdam UMC University of Amsterdam Amsterdam the Netherlands; ^3^ Department of Surgery, Cancer Centre Amsterdam, Amsterdam UMC VU University Amsterdam the Netherlands; ^4^ Department of Radiology, Amsterdam UMC VU University Amsterdam the Netherlands; ^5^ Department of Radiotherapy Amsterdam the Netherlands; ^6^ Department of Radiology Netherlands Cancer Institute Amsterdam the Netherlands; ^7^ Department of Surgery Zwolle the Netherlands; ^8^ Department of Radiotherapy Isala Hospital Zwolle the Netherlands; ^9^ Department of Research Netherlands Comprehensive Cancer Organization Utrecht the Netherlands; ^10^ Department of Gastroenterology and Hepatology University Medical Centre Utrecht Utrecht the Netherlands; ^11^ Department of Surgery Reinier de Graaf Hospital Delft the Netherlands

## Abstract

**Background:**

Adequate MRI‐based staging of early rectal cancers is essential for decision‐making in an era of organ‐conserving treatment approaches. The aim of this population‐based study was to determine the accuracy of routine daily MRI staging of early rectal cancer, whether or not combined with endorectal ultrasonography (ERUS).

**Methods:**

Patients with cT1–2 rectal cancer who underwent local excision or total mesorectal excision (TME) without downsizing (chemo)radiotherapy between 1 January 2011 and 31 December 2018 were selected from the Dutch ColoRectal Audit. The accuracy of imaging was expressed as sensitivity, specificity, and positive predictive value (PPV) and negative predictive value.

**Results:**

Of 7382 registered patients with cT1–2 rectal cancer, 5539 were included (5288 MRI alone, 251 MRI and ERUS; 1059 cT1 and 4480 cT2). Among patients with pT1 tumours, 54·7 per cent (792 of 1448) were overstaged by MRI alone, and 31·0 per cent (36 of 116) by MRI and ERUS. Understaging of pT2 disease occurred in 8·2 per cent (197 of 2388) and 27·9 per cent (31 of 111) respectively. MRI alone overstaged pN0 in 17·3 per cent (570 of 3303) and the PPV for assignment of cN0 category was 76·3 per cent (2733 of 3583). Of 834 patients with pT1 N0 disease, potentially suitable for local excision, tumours in 253 patients (30·3 per cent) were staged correctly as cT1 N0, whereas 484 (58·0 per cent) and 97 (11·6 per cent) were overstaged as cT2 N0 and cT1–2 N1 respectively.

**Conclusion:**

This Dutch population‐based analysis of patients who underwent local excision or TME surgery for cT1–2 rectal cancer based on preoperative MRI staging revealed substantial overstaging, indicating the weaknesses of MRI and missed opportunities for organ preservation strategies.

## Graphical abstract

This population‐based study determined the accuracy of routine daily MRI staging of early rectal cancer, whether or not combined with endorectal ultrasonography. The results showed that preoperative MRI led to substantial overstaging in patients who underwent local excision or total mesorectal incision surgery for cT1–2 rectal cancer, indicating the weaknesses of MRI and missed opportunities for organ preservation strategies.

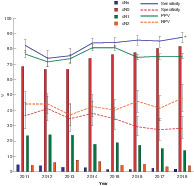

Imaging weakness

## Abstract (es)

**Antecedentes:**

Una adecuada estadificación mediante resonancia magnética nuclear (RMN) de los cánceres de recto en estadios precoces es esencial para la toma de decisiones en una era en la existen diferentes opciones de tratamiento preservadoras del recto. El objetivo de este estudio de base poblacional fue determinar la precisión de la estadificación mediante RMN del cáncer de recto precoz en la práctica diaria, ya sea combinada o no con la ecografía endorectal (*endorectal ultrasound*, ERUS).

**Métodos:**

Los pacientes con cáncer de recto en estadio cT1‐2 que se sometieron a resección local o resección total del mesorrecto (*total mesorectal excision*, TME) sin (quimio) radioterapia neoadyuvante fueron seleccionados a partir del registro auditado ColoRectal holandés, entre el 1 de enero de 2011 y el 31 de diciembre de 2018. La precisión de las imágenes se expresó como sensibilidad, especificidad y valores predictivos positivo y negativo (*positive‐ and negative predicting value*, PPV / NPV).

**Resultados:**

De un total de 7.382 pacientes registrados con cáncer de recto en estadio cT1‐2, se incluyeron 5.539 pacientes (5.288 solamente RMN, 251 RMN + ERUS; 1.059 cT1 y 4.480 cT2). Los pacientes pT1 fueron sobreestadificados cuando se utilizó únicamente la RMN en un 54,7% de los casos (792/1.448) y cuando se combinó RMN y ERUS en un 31,0% (36/116). La infraestadificación de pT2 ocurrió en un 8,2% (197/2.388) y en un 27,9% (31/111), respectivamente. La RMN utilizada como única prueba sobreestadificó los casos pN0 en el 17,3% (570/3.303) y el VPP del estadio cN0 fue del 76,3% (2.733/3.583). De los 834 pacientes con estadio pT1N0, potencialmente adecuado para la resección local, 253 pacientes (30,3%) se clasificaron correctamente como cT1N0, y 484 (58,8%) y 97 (11,6%) pacientes se sobreestadificaron como cT2N0 y cT1‐2N1, respectivamente.

**Conclusión:**

Este estudio de base poblacional holandés en pacientes que se sometieron a una resección local o a cirugía TME por cáncer de recto cT1‐2 con estadificación preoperatoria mediante RMN, muestra una considerable sobreestadificación, lo que indica las debilidades y oportunidades en las estrategias de preservación del recto.

## Introduction

Rectal cancer management depends on clinical locoregional staging by endoscopy, endorectal ultrasonography (ERUS) and MRI. The decision whether to treat rectal cancer either by rectum‐sparing local excision or radical excision, with or without the addition of preoperative (chemo)radiotherapy ((C)RT), is dictated by clinical staging and identification of risk factors such as T category, suspicious lymph nodes and extramural venous invasion^1^. The use of MRI for determining indication for preoperative radiotherapy and extent of subsequent rectal resection in patients with intermediate‐ or high‐risk rectal cancer has significantly influenced rectal cancer care worldwide^2^.

The introduction of bowel cancer screening programmes has resulted in stage migration, with an increase in early‐stage rectal cancer (cT1–2 N0)^3^. This opens opportunities for organ preservation, avoiding substantial morbidity and decreased function associated with radical rectal resection^4^. However, correct patient selection with optimal staging is a prerequisite for a safe organ preservation strategy^4^. Superficial lesions (T1) without risk features and/or suspicious lymph nodes might be considered suitable for upfront local excision as part of an organ‐preserving strategy. However, the final pathology may reveal more advanced tumour stage or the presence of adverse features leading to completion total mesorectal excision (TME) or adjuvant CRT within current trials^5^, ^6^. Another approach aimed at organ preservation for patients with early cancer is upfront (C)RT with subsequent response assessment and tailored adjuvant treatment (such as TME surgery, local excision, watch‐and‐wait strategy) within current trials. Although this approach is promising, a substantial proportion (about 50 per cent) of patients are at risk of overtreatment, especially given the current limitations of clinical staging^7^, ^8^.

To avoid both undertreatment and overtreatment, optimizing clinical staging is of utmost importance. MRI‐based staging of rectal cancer has proven value in more advanced stages of the disease, but is known for its limited accuracy in early rectal cancers and assessment of lymph node status^9^. The diagnostic value largely depends on MRI protocols and experience of the radiologist. For early lesions, the addition of ERUS has been advocated to improve clinical staging, but this seems highly operator‐dependent^10^. At present, population‐based data on the accuracy of clinical tumour and nodal staging of early rectal cancer are lacking. Therefore, the aim of this population‐based study was to determine the diagnostic accuracy of routine MRI staging, with or without ERUS, in patients who underwent local excision or TME surgery for cT1–2 rectal cancer.

## Methods

Data were derived from the Dutch ColoRectal Audit (DCRA). This audit collects detailed information on patient, tumour and treatment characteristics, and short‐term outcomes (within 90 days) from all patients undergoing resection for primary colorectal cancer in the Netherlands. Specific details of the DCRA regarding data collection, data quality, data validation and methodology have been published previously^11^.

### Patient selection

All patients who underwent local excision or TME surgery for primary cT1–2 rectal cancer, and were registered in the DCRA between 1 January 2011 and 31 December 2018, were potentially eligible. Only patients who were staged by MRI, with or without ERUS, were included. Exclusion criteria were: downsizing therapy (such as short‐course radiotherapy (SCRT) with delayed surgery or CRT), a registered (y)pT0 tumour, emergency surgery, and staging by modalities other than MRI with or without ERUS. Patients who underwent SCRT with immediate surgery (interval to surgery 2 weeks or less) were considered eligible. For this study, no ethical approval or informed consent was required under Dutch law.

### Data extraction and outcome parameters

The following data were extracted from the DCRA database: patient and tumour characteristics, and diagnostic, staging and procedural data. Data on ERUS were available in the DCRA data set until 2017. Removal of this variable was related to the low uptake of ERUS at a national level and registration burden. Pathological T and N categories were extracted as the standard for comparison with radiological staging. Understaging of patients with rectal cancer was defined by a higher pTN compared with cTN category, and overstaging by a lower pTN compared with cTN category.

Outcome parameters included sensitivity, specificity, positive predictive value (PPV) and negative predictive value (NPV) of preoperative MRI in determining tumour and node categories.

### Dutch colorectal cancer guidelines

The Dutch colorectal cancer guideline of 2008^12^ was revised in 2014^13^. To determine the cT category of rectal cancer, both Dutch guidelines recommended MRI as part of the standard evaluation of non‐superficial tumours. ERUS can be considered in addition to MRI for differentiating T1 from T2 disease. Regarding nodal staging, the Dutch guideline of 2008^12^ stated that lymph nodes larger than 5 mm on MRI should be considered suspicious for nodal metastasis. The revised 2014 guideline^13^ stated that nodal disease should be considered in lymph nodes with a size of 5–9 mm and the presence of at least two of three malignant morphological characteristics, or in lymph nodes measuring more than 9 mm.

### Statistical analysis

Baseline characteristics are described as numbers with percentages. Staging was evaluated for patients who underwent MRI alone, and those who had combined imaging by MRI and ERUS. To calculate the diagnostic performance of imaging, cT and cN categories were compared with pT and pN categories. Both local excisions and TME were included in the analysis of T category, whereas only TME was included in the analysis of N category. Sensitivity, specificity, PPV and NPV were calculated, with 95 per cent confidence intervals, for analyses of cT1, cT2, cN0, cN1 and cN2. These outcome parameters were also calculated by year, and analysed for time trends using the linear‐by‐linear association test for the total study interval (2011–2018), and comparing two time periods (before 2014 *versus* 2014 onwards) to explore the impact of revision of the national colorectal cancer guideline in 2014. Regarding N category, nodal positivity (N+) was used without discriminating N1 from N2. *P* < 0·050 was considered statistically significant. Statistical analyses were performed in SPSS® for Windows® version 24.0 (IBM, Armonk, New York, USA).

## Results

A total of 7382 patients with cT1–2 rectal cancer were identified between 2011 and 2018. Patient selection is shown in *Fig*. *S1* (supporting information). During the study interval, the use of MRI for clinical staging in this population increased from 90·4 per cent in 2011 to 92·6 per cent in 2018. A total of 5539 patients remained for final analysis after exclusion of those who had preoperative (C)RT with an interval of more than 2 weeks to surgery, those treated in an emergency setting, and patients whose tumours were staged by means other than MRI with or without ERUS. Staging was performed by MRI alone in 5288 patients, and combined imaging by MRI and ERUS in 251. The use of ERUS in combination with MRI increased from 0·2 per cent in 2011 to 5·4 per cent in 2017. Some 1059 patients (19·1 per cent) had cT1 and 4480 (80·9 per cent) had cT2 disease.

Patient, tumour and treatment characteristics of the included patients are shown in *Table* 1, stratified by type of surgery. The median number of lymph nodes examined increased from 11 in 2011 to 15 in 2018 (*Fig*. *S2*, supporting information). The proportion of pN+ disease remained similar over time (median 28 per cent), as well as the total number of positive lymph nodes in patients with pN+ disease (median 2).

**Table 1:** Patient, tumour and treatment characteristics of patients with cT1–2 rectal cancer diagnosed by MRI with or without endorectal ultrasonography between 2011 and 2018, who subsequently underwent local excision or total mesorectal excision without downsizing preoperative radiotherapy

		TME surgery (*n* = 4847)	Local excision (*n* = 692)
**Age (years)**	< 75	3508 (72·4)	497 (71·9)
	≥ 75	1336 (27·6)	194 (28·1)
	Missing	3	1
**Sex**	M	2997 (61·9)	461 (66·6)
	F	1848 (38·1)	231 (33·4)
	Missing	2	0
**ASA fitness grade**	I–II	4000 (82·5)	532 (76·9)
	≥ III	846 (17·5)	160 (23·1)
	Missing	1	0
**Charlson score**	0	2619 (54·0)	349 (50·4)
	1	1044 (21·5)	166 (24·0)
	≥ 2	1184 (24·4)	177 (25·6)
**BMI (kg/m** ^**2**^ **)**	< 30	3889 (81·6)	578 (84·0)
	≥ 30	879 (18·4)	110 (16·0)
	Missing	79	4
**Preoperative MRI staging**	MRI alone	4700 (97·0)	588 (85·0)
	MRI including ERUS	147 (3·0)	104 (15·0)
Tumour distance from anus (cm)*	≤ 5	1564 (33·7)	354 (56·0)
	6–10	1820 (39·3)	224 (35·4)
	≥ 10	1252 (27·0)	54 (8·5)
	Missing	211	60
**Clinical tumour category**	cT1	577 (11·9)	482 (69·7)
	cT2	4270 (88·1)	210 (30·3)
**Clinical node category**	cN0	3746 (77·4)	–
	cN1	942 (19·5)	–
	cN2	81 (1·7)	–
	cNx/unknown	69 (1·4)	–
	Missing	9	
**Clinical metastasis category**	cM0	4542 (93·9)	–
	cM1	82 (1·7)	–
	cMx/unknown	215 (4·4)	–
	Missing	8	
**Neoadjuvant radiotherapy**	None	3227 (66·6)	689 (99·6)
	SCRT‐IS	1620 (33·4)	3 (0·4)
**Surgical procedure**	(L)AR	3115 (64·3)	–
	APR	1044 (21·5)	–
	Hartmann	578 (11·9)	–
	Other†	110 (2·3)	–
**Pathological tumour category**	pT1	1057 (21·8)	507 (73·3)
	pT2	2331 (48·1)	168 (24·3)
	pT3	1403 (28·9)	17 (2·5)
	pT4	56 (1·2)	0 (0)
**Pathological node category**	pN0	3465 (71·5)	–
	pN1	1046 (21·6)	–
	pN2	294 (6·1)	–
	pNx/unknown	42 (0·9)	–
**Circumferential resection margin**	Positive (≤ 1 mm)	119 (2·7)	–
	Negative	4222 (97·3)	–
	Missing	506	
**No. of lymph nodes retrieved**	≤ 10	1060 (21·9)	–
	> 10	3779 (78·0)	–
	Unknown	8 (0·2)	
**No. of positive lymph nodes**	0	3492 (72·1)	–
	1–3	1034 (21·4)	
	> 3	317 (6·5)	–
	Missing	4	–

Values in parentheses are percentages, excluding missing values. *Not defined in Dutch ColoRectal Audit until 2016 and mostly based on endoscopic measurement of distance to anal verge; since 2016 defined as distance to anorectal junction on sagittal MRI. †Includes proctocolectomy and total colectomy. TME, total mesorectal excision; ERUS, endorectal ultrasonography; SCRT‐IS, short‐course radiotherapy–immediate surgery (within 2 weeks); (L)AR, low anterior resection; APR, abdominoperineal resection.

### Diagnostic performance for tumour category

The diagnostic performance of preoperative MRI alone and with the addition of ERUS was assessed by tumour and node category (*Tables* 2, 3, 4, 5, 6). Of 942 patients with cT1 disease, 656 (69·6 per cent) had pT1, 197 (20·9 per cent) had pT2 and 85 (9·0 per cent) had pT3 tumours. Of 4346 patients with cT2 disease, 792 (18·2 per cent) had pT1, 2191 (50·4 per cent) had pT2 and 1311 (30·2 per cent) had pT3 tumours (*Table* 2). Overstaging of pT1 tumours occurred in 54·7 per cent and understaging of pT2 tumours in 8·2 per cent. The sensitivity and specificity of MRI alone were 45·3 and 92·6 per cent respectively for T1 tumours, and 91·8 and 25·7 per cent for T2 tumours (*Table* 7). Overall, assignment of cT1–2 category of rectal cancer by MRI alone was accurate in 2847 patients (53·8 per cent); 792 patients (15·0 per cent) were overstaged clinically in terms of category, and 1649 (31·2 per cent) were understaged clinically. Combining the results for cT1–2 tumours evaluated by MRI alone, 26·4 per cent were pT3 and 1·1 per cent were pT4 lesions.

**Table 2:** Clinical *versus* pathological tumour category assignment by MRI alone, including local excisions

	pT1	pT2	pT3	pT4	Total
cT1	656	197	85	4	942
cT2	792	2191	1311	52	4346
Total	1448	2388	1396	56	5288

**Table 3:** Clinical *versus* pathological nodal staging by MRI alone

	pN0	pN1	pN2	pNx	Total
cN0	2733	672	178	32	3615
cN1	524	311	89	6	930
cN2	46	15	18	0	79
cNx	36	24	3	4	67
Total	3339	1022	288	42	4691*

* cN/pN was missing for nine patients.

**Table 4:** Clinical *versus* pathological tumour category assignment by MRI and endorectal ultrasonography, including local excisions

	pT1	pT2	pT3	Total
cT1	80	31	6	117
cT2	36	80	18	134
Total	116	111	24	251

**Table 5:** Clinical *versus* pathological nodal staging by MRI and endorectal ultrasonography

	pN0	pN1	pN2	Total
cN0	106	22	3	131
cN1	10	1	1	12
cN2	1	0	1	2
cNx	2	0	0	2
Total	119	23	5	147

**Table 6:** Diagnostic accuracy of clinical staging of combined T and N category by MRI alone

	pT1 N0	pT2 N0	pT1–2 N1	Total
cT1 N0	253	87	47	387
cT2 N0	484	1312	289	2085
cT1–2 N1	97	279	177	553
Total	834	1678	513	3025*

* Tumours were staged as ‘other’ in 1675 patients (35·6 per cent).

**Table 7:** Accuracy of MRI alone for tumour and node staging

	Sensitivity (%)	Specificity (%)	PPV (%)	NPV (%)	Accuracy (%)
**Tumour category, including local excisions**					
cT1	45·3 (42·7, 47·9)	92·6 (91·7, 93·4)	69·6 (66·9, 72·2)	81·8 (81·1, 82·5)	79·6 (78·5, 80·7)
cT2	91·8 (90·6, 92·8)	25·7 (24·1, 27·3)	50·4 (49·8, 51·0)	79·1 (76·5, 81·4)	55·5 (54·2, 56·9)
**Node category**					
cN0	82·7 (81·4, 84·0)	33·7 (31·2, 36·4)	76·3 (75·5, 77·0)	43·2 (40·6, 45·8)	69·0 (67·7, 70·4)
cN1	31·2 (28·3, 34·1)	82·9 (81·6, 84·1)	33·7 (31·1, 36·3)	81·2 (80·6, 81·9)	71·7 (70·3, 73·0)
cN2	6·3 (3·8, 9·8)	98·6 (98·2, 98·9)	22·8 (15·0, 33·0)	94·1 (93·9, 94·2)	92·9 (92·1, 93·6)

Values in parentheses are 95 per cent confidence intervals. PPV, positive predictive value; NPV, negative predictive value.

In patients who were staged by both MRI and ERUS, overstaging of pT1 occurred in 31·0 per cent (36 of 116) and understaging of pT2 in 27·9 per cent (31 of 111) (*Table* 4). The sensitivity and specificity of combined MRI and ERUS was 69·0 and 72·6 per cent respectively for T1 tumours, with corresponding values of 72·1 and 61·4 per cent for T2 tumours (*Table* 8).

**Table 8:** Accuracy of MRI and endorectal ultrasonography for tumour and node staging

	Sensitivity (%)	Specificity (%)	PPV (%)	NPV (%)	Accuracy (%)
**Tumour category, including local excisions**					
cT1	69·0 (59·7, 77·2)	72·6 (64·3, 79·9)	68·4 (61·6, 74·5)	73·1 (67·1, 78·5)	70·9 (64·9, 76·5)
cT2	72·1 (62·8, 80·2)	61·4 (52·8, 69·5)	59·7 (53·8, 65·3)	73·5 (66·7, 79·4)	66·1 (59·9, 72·0)
**Node category**					
cN0	90·6 (83·8, 95·2)	10·7 (2·3, 28·2)	80·9 (78·6, 83·0)	21·4 (7·5, 47·7)	75·2 (67·3, 82·0)
cN1	4·3 (0·1, 22·0)	91·0 (84·4, 95·4)	8·3 (1·2, 40·1)	83·5 (82·0, 84·8)	77·2 (69·6, 83·8)
cN2	20·0 (0·5, 71·6)	99·3 (96·1, 99·9)	50·0 (6·8, 93·2)	97·2 (95·7, 98·2)	96·6 (92·1, 98·9)

Values in parentheses are 95 per cent confidence intervals. PPV, positive predictive value; NPV, negative predictive value.

**Figure 1:**
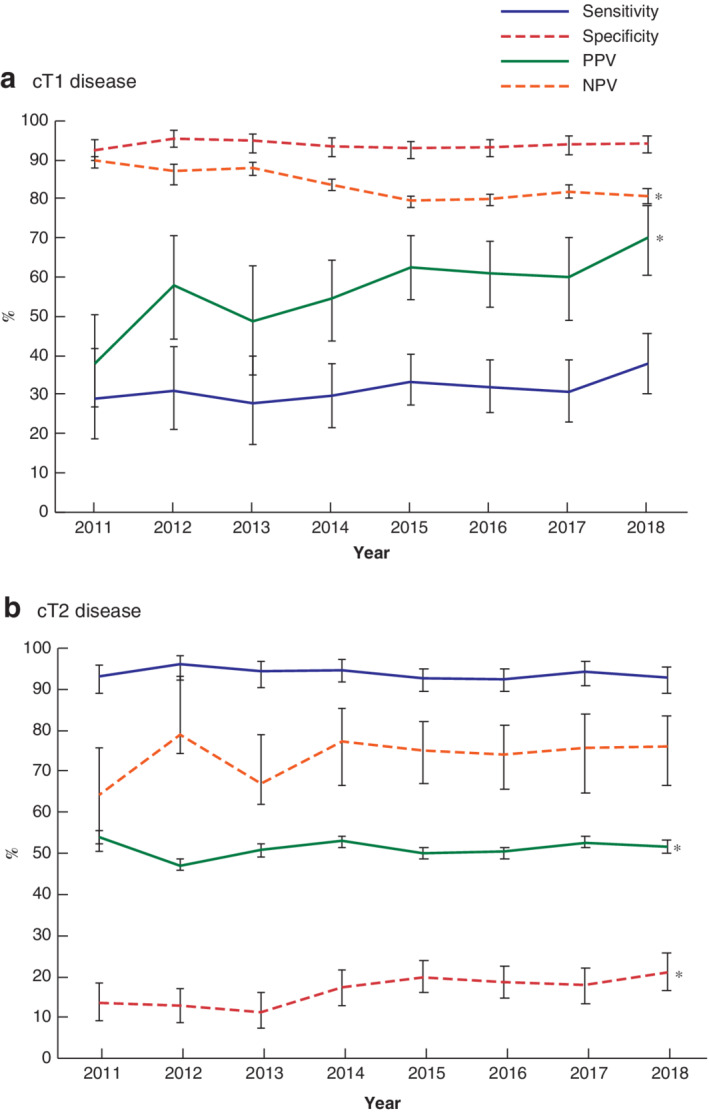
Diagnostic indices of MRI for diagnosis of cT1 and cT2 rectal cancer over time, 2011–2018 **a** cT1 and **b** cT2 disease. Sensitivity, specificity, positive predictive value (PPV) and negative predictive value (NPV) are shown with 95 per cent intervals. *P < 0·050 over total study interval (linear‐by‐linear association test).

Trends in sensitivity, specificity, PPV and NPV by individual clinical tumour category over the study interval are shown in *Fig*. 1. The PPV of MRI for staging cT1 tumours showed a significant increase of 32·1 per cent between 2011 and 2018 (*P* < 0·050). NPV decreased significantly from 89·6 to 80·7 per cent in same interval (*P* < 0·050) (*Fig*. 1 *a*). For cT2 tumours, the specificity showed a significant increase of 9·4 per cent from 2013 to 2018 (*P* < 0·050) and there was a significant increase in PPV of 4·5 per cent from 2012 to 2018 (*P* < 0·050) (*Fig*. 1 *b*).

### Assessment of tumour category for local excisions

In patients who were staged by MRI alone and underwent local excision, overstaging of pT1 occurred in 23·5 per cent (100 of 426) and understaging of pT2 in 45·2 per cent (66 of 146) (*Table* *S1*, supporting information). For the local excision group, the sensitivity and specificity of MRI alone were 76·5 and 56·2 per cent respectively for T1 tumours, and 54·8 and 74·9 per cent for T2 tumours (*Table S2*, supporting information).

For patients who underwent local excision and were staged by MRI and ERUS, overstaging of pT1 occurred in 12 per cent (10 of 81) and understaging of pT2 in 59 per cent (13 of 22) (*Table S3*, supporting information). The sensitivity and specificity of MRI and ERUS combined were 87·7 and 39·1 per cent respectively for T1 tumours, and 40·9 and 87·8 per cent for T2 tumours (*Table S4*, supporting information).

### Diagnostic performance for node category

Preoperative assessment of node category was available for 4769 patients who had TME (98·4 per cent). The proportion of patients with missing cN category in the population decreased from 3·5 per cent in 2011 to 0·7 per cent in 2018. Assessment of overall node category was accurate in 3066 patients (65·4 per cent) by MRI alone; nodal disease was understaged clinically in 977 patients (20·8 per cent) and overstaged clinically in 570 (12·2 per cent) (*Table* 3). Using MRI alone, the accuracy for cN0 category was 69·0 per cent, with a sensitivity of 82·7 per cent and specificity of 33·8 per cent (*Table* 7). Overstaging of pN0 disease occurred in 17·3 per cent (570 of 3303), and understaging of pN1–2 in 66·3 per cent (850 of 1283).

Tumours in 131 patients were assigned as cN0 by combined MRI and ERUS, of which 25 were pN1–2 (19·1 per cent) (*Table* 5). The accuracy for cN0 category by MRI and ERUS was 75·2 per cent, with a sensitivity of 90·6 per cent and specificity of 10·7 per cent (*Table* 8).

**Figure 2:**
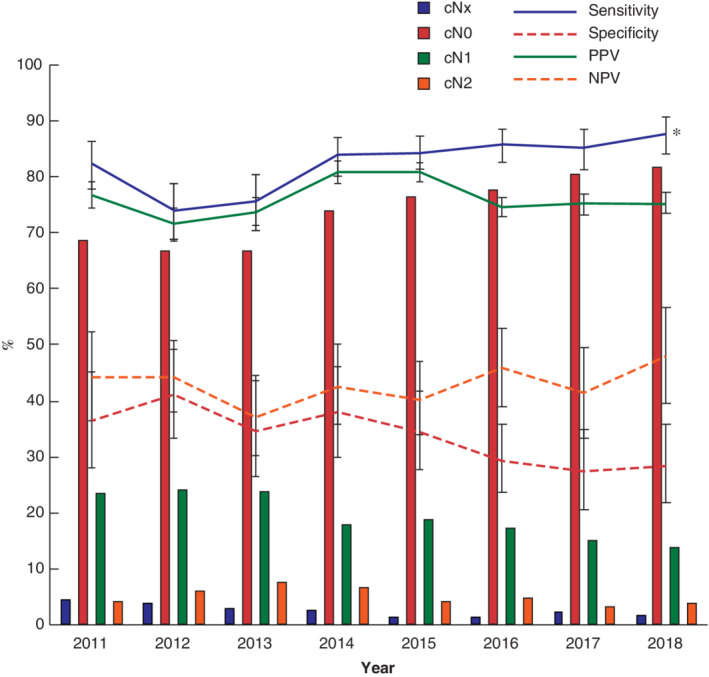
Clinical node categories and diagnostic indices for clinical mesorectal lymph node assignment based on MRI (cN0 and cN1–2 *versus* pN0 and pN1–2) in patients with cT1–2 rectal cancer based on MRI for each year, 2011–2018 Clinical node category is shown for all patients who underwent surgical resection of rectal cancer. Sensitivity, specificity, positive predictive value (PPV) and negative predictive value (NPV) are shown with 95 per cent intervals. *P < 0·050 over total study interval (linear‐by‐linear association test).

In this population of patients with cT1–2 rectal cancer assessed by MRI who underwent TME, there was a decreasing trend in diagnosis of cN1–2 disease after revision of the guideline in 2014 (*Fig*. 2). The sensitivity for determination of node category by MRI showed a significant increase from 74·0 per cent in 2012 to 87·6 per cent in 2018 (*P* < 0·050). A significant decrease in PPV was observed from 80·8 per cent in 2014 to 75·3 per cent in 2018 (*P* < 0·050). Additional time‐trend analysis showed a significant decrease in specificity over the total study period (*P* = 0·031) and between the two time intervals (before 2014 *versus* 2014 onwards) (*P* = 0·039).

### Diagnostic accuracy of combined tumour and node staging by MRI

The clinically relevant combined staging according to T and N category was evaluated (*Table* 6). Among 834 patients with pT1 N0 disease, potentially suitable for local excision only, tumours were correctly staged as cT1 N0 in 253 (30·3 per cent), but overstaged as cT2 N0 in 484 patients (58·0 per cent) and cT1–2 N1 in 97 patients (11·6 per cent). Eighty‐seven of 1678 pT2 N0 tumours (5·2 per cent) and 47 of 513 pT1–2 N1 tumours (9·2 per cent) were staged incorrectly as cT1 N0. Of 513 patients with pT1–2 N1 disease, 177 (34·5 per cent) were correctly staged by MRI alone.

## Discussion

In this Dutch population‐based study, among the 7382 patients with a registered cT1–2 rectal cancer treated between 2011 and 2018, 9·4 and 65·7 per cent of patients underwent local excision and TME respectively after MRI staging without downstaging preoperative therapy. In this combined population comprising a total of 5539 patients, 4·5 per cent also underwent ERUS for preoperative staging. The overall performance of MRI with or without ERUS in staging early rectal cancer was disappointing. The diagnostic value of preoperative MRI showed some improvements in tumour and nodal staging over time, but potential areas for improvement remain. Of tumours assigned as cT1 on MRI‐based preoperative clinical assessment, 69·6 per cent were pT1 and 20·9 per cent were pT2 lesions; 8·2 per cent of pT2 cancers were understaged. Most striking was the overstaging of pT1 tumours with MRI alone (54·7 per cent) and MRI plus ERUS (31·0 per cent). The accuracy of cN0 staging was 69·0 per cent; overstaging of pN0 disease occurred in 17·3 per cent, and understaging of pN1–2 in 66·3 per cent. Of pT1 N0 tumours in 834 patients, only 253 (30·3 per cent) were correctly staged by MRI as cT1 N0; in 484 patients (58·0 per cent) the disease was overstaged as cT2 N0, limiting the use of upfront local excision.

In the present study, the diagnostic value of MRI for tumour and nodal staging was determined specifically for early tumour stages. Most studies reporting on the diagnostic performance of MRI included all stages of rectal cancer. The meta‐analyses of Al‐Sukhni and colleagues^14^, including 21 studies, and Bipat *et al*.^15^, including 90 studies, demonstrated overall sensitivities ranging from 69 to 87 per cent, and specificities of 75–82 per cent for determination of T category by MRI. Patients with rectal cancer who underwent preoperative downsizing therapy were excluded from the meta‐analysis reported by Zhang and co‐workers^16^ comprising a total of 35 studies. Subgroup analyses for T1 and T2 tumours showed sensitivities of 58 and 80 per cent respectively (45·3 and 91·8 per cent in the present study) and specificities of 97 and 74 per cent (92·6 and 25·7 per cent here). In accordance with the present findings, a high specificity for cT1 and a high sensitivity for cT2 disease were reported.

The diagnostic value of nodal staging by MRI remains a subject of debate. The revised Dutch colorectal cancer guideline from 2014^13^ specifically included stricter criteria for lymph node positivity on MRI. Remarkably, a significant decrease in specificity for nodal staging was observed from 2014 (*P* = 0·039), meaning that an increased tendency toward overstaging was observed over time in this population of early rectal cancers, despite current guideline recommendations. In a study^17^ of 52 patients with T1–3 rectal cancer, preoperative nodal staging by MRI had an accuracy of 60 per cent, sensitivity of 57 per cent and specificity of 83 per cent. In another study^18^ that evaluated 65 early rectal cancers, the accuracy was 84 per cent, with a PPV of 71 per cent and NPV of 90 per cent for MRI‐based nodal staging. The present study confirmed the overstaging by MRI for nodal disease in this specific group of patients with early tumours, in which 56·3 per cent of cN1 tumours were pN0. A possible explanation for overstaging might be based on the use of size criteria for nodal staging and hospital variation in adherence to guidelines. Overstaging of positive nodal disease with use of over 5 mm as a size criterion, which was a recommendation in the colorectal cancer guideline of 2008^12^, has been reported in 30–40 per cent^19^. In the TESAR trial^6^, which is investigating therapeutic options after local excision of early rectal cancer with risk features, lymph nodes smaller than 10 mm are considered benign independent of morphological features.

Radiologists have a learning curve to become proficient in staging of early rectal cancer by MRI. Rafaelsen and colleagues^20^ reported a higher sensitivity (96 *versus* 77 per cent; *P* < 0·050) and specificity (74 *versus* 40 per cent; *P* < 0·050) for more experienced gastrointestinal radiologists compared with general radiologists in assessing all stages of rectal cancer. This implies that specific training programmes and accreditation for radiologists is likely to improve the accuracy of early rectal cancer staging^21^. Furthermore, tumour staging improves with the use of higher field strength MRI and review of images ideally by consensus of two or more expert radiologists^16^, ^17^. Recently, the national SPECC (significant polyps and early colorectal cancer) initiative in the UK expressed the need to improve staging of early rectal cancer, including better focus on standardization of MRI protocols, consensus guidelines, (size) criteria used for nodal staging, structured MRI reporting, and increasing performance and experience in smaller centres^4^, ^21^.

The European Society of Gastrointestinal and Abdominal Radiology consensus document explicitly mentions the role of (additional) ERUS, given its superior diagnostic performance in differentiating T1 from T2 tumours^22^. In the present study, the addition of ERUS only slightly improved nodal staging in comparison with MRI alone, and showed overstaging of pT1 and understaging of pT2, but numbers were small because of the restricted use of ERUS in the Netherlands. As these modalities are complementary to each other, the reported accuracy should be interpreted as such, and not as the sole accuracy of MRI or ERUS. A recent study^23^ showed that ERUS outperformed MRI in overall T, T1, T3 and overall nodal staging (*P* < 0·010). These results support the suggestion that ERUS is better at detecting smaller lesions in the thinner colorectal wall (submucosa and serosa) in contrast to the muscularis propria (T2), at which level MRI performs better. However, Mondal and co‐workers^24^ showed no benefit of ERUS in patient selection for local therapy, with less than 10 per cent change in management when results of ERUS were considered alongside clinical, endoscopic and MRI staging findings.

Given the diagnostic difficulties in MRI staging as found in the present analysis, upfront CRT strategies carry a risk of overtreatment as many small T1 lesions will be included that could be treated with local excision only. The potential of upfront (C)RT in organ preservation is currently being investigated in an international controlled trial^7^, ^25^. Another more pragmatic strategy aimed at organ preservation for cT1–2 tumours of limited size is diagnostic/therapeutic local excision. Exact histopathology will reveal the final staging and, for the majority, radical local excision with endoscopic submucosal dissection (ESD) or transanal minimally invasive surgery (TAMIS) already constitutes definitive treatment for low‐risk T1 tumours. If the final pathology shows an intermediate‐risk early cancer (high‐risk pT1 or low‐risk pT2), additional treatment options should be considered: completion TME surgery, adjuvant CRT, or close surveillance including MRI for local and/or nodal regrowth. However, the last two of these options are still considered experimental. Implementing diagnostic/therapeutic local excision by ESD or TAMIS for all cT1–2 tumours requires patient as well as surgeon and multidisciplinary team education. Patients should be aware of the possibility that completion TME is advised if the final pathology reveals high‐risk features. However, completion TME is regarded as a slightly riskier procedure because it is associated with higher morbidity and colostomy rates, and risk of incomplete specimens, compared with primary TME^26^. However, these increased risks seem less evident with transanal TME^27^. In addition, the potential problems of subsequent completion surgery should be considered before deciding on local excision, and full‐thickness excisions should be avoided in areas at risk of breach of completion TME surgery (anterior rectum and close to sphincter). Alternative therapeutic approaches, adjuvant CRT and close surveillance with endoscopy and MRI are experimental arms in the ongoing TESAR trial^6^, ^25^.

The strength of this population‐based study is that it provides real‐life data concerning the diagnostic accuracy of preoperative MRI staging of early rectal cancer in daily practice in the Netherlands. However, some limitations need to be addressed. Patients who underwent endoscopic polypectomy of a T1 cancer, or were referred to dedicated units in pursuit of a rectum‐preserving strategy were not included, which might have influenced the overall diagnostic accuracy of MRI and ERUS. The decision‐making process regarding the use of additional ERUS for staging was not registered in the DCRA data set. Furthermore, no data were available regarding the different MRI protocols used to stage rectal cancer across the centres. Furthermore, the variables currently available in the DCRA do not allow analyses of the percentage of patients who could eventually have been spared TME surgery based on all currently known high‐risk features available to guide this decision, or who needed completion TME if routine upfront local excision had been implemented.

This large population‐based study demonstrated the diagnostic value of preoperative MRI in patients who underwent local excision or TME surgery for early rectal cancer in routine daily practice in the Netherlands between 2011 and 2018. It revealed a substantial rate of overstaging of pT1 N0 tumours, which eliminated the option of an organ‐preserving approach instead of TME surgery in eligible patients.

## Collaborators

Members of the Dutch ColoRectal Audit Group: A. G. J. Aalbers (Department of Surgery, Netherlands Cancer Institute–Antoni van Leeuwenhoek, Amsterdam); W. A. Bemelman (Department of Surgery, Amsterdam UMC, location AMC, Amsterdam); F. C. den Boer (Department of Surgery, Zaans Medisch Centrum, Zaandam); S. O. Breukink (Department of Surgery, MUMC, Maastricht); P. P. L. O. Coene (Department of Surgery, Maasstad Ziekenhuis, Rotterdam); P. G. Doornebosch (Department of Surgery, IJsselland Ziekenhuis, Capelle aan den IJssel); J. W. de Groot (Department of Oncology, Isala Hospital, Zwolle); T. M. Karsten (Department of Surgery, OLVG, Amsterdam); M. Ledeboer (Department of Gastroenterology and Hepatology, Deventer Ziekenhuis, Deventer); E. R. Manusama (Department of Surgery, Medical Centre Leeuwarden, Leeuwarden); I. D. Nagtegaal (Department of Pathology, Radboudumc, Nijmegen); K. C. M. J. Peeters, R. A. E. M. Tollenaar, C. J. H. van de Velde (Department of Surgery, LUMC, Leiden); A. Wagner (Department of Clinical Genetics, Erasmus MC, Rotterdam); M. Westerterp (Department of Surgery, MC Haaglanden, Den Haag).

## Supporting information

Fig. S1 Study flow chart

Fig. S2 Numbers of lymph nodes over time

Table S1 Clinical *versus* pathological tumour staging by MRI alone in local excisions
Table S2 Accuracy of MRI alone in tumour staging local excisions
Table S3 Clinical *versus* pathological tumour staging by MRI + ERUS in local excisions
Table S4 Accuracy of MRI + ERUS in tumour staging local excisions
