# Supplementary figures and images for: MRI cT1–2 rectal cancer staging accuracy: a population‐based study
Source: Br J Surg. 2020 Apr 16;107(10):1372–82. doi: 10.1002/bjs.11590 (PMC7496930; doi:10.1002/bjs.11590)

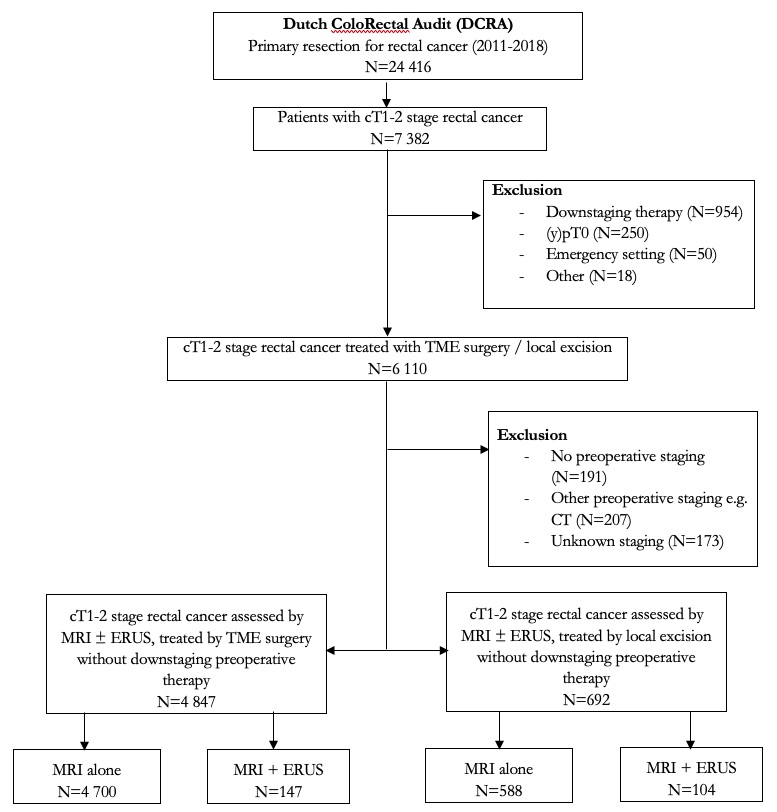

Supplement: Supplementary file 1 — Fig. S1 Study flow chart [file BJS-107-1372-s001.jpg]

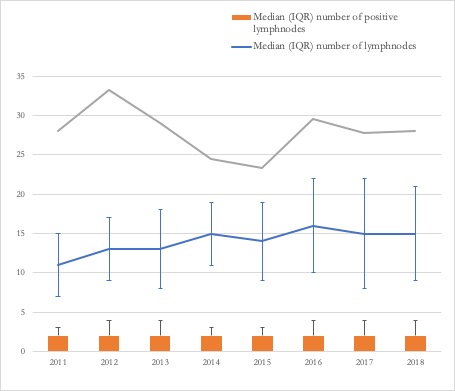

Supplement: Supplementary file 2 — Fig. S2 Numbers of lymph nodes over time [file BJS-107-1372-s002.jpg]
