# Supplementary material for: MRI cT1–2 rectal cancer staging accuracy: a population‐based study
Source: Br J Surg. 2020 Apr 16;107(10):1372–82. doi: 10.1002/bjs.11590 (PMC7496930; doi:10.1002/bjs.11590)
Supplement: Supplementary file 3 — Table S1 Clinical versus pathological tumour staging by MRI alone in local excisions Table S2 Accuracy of MRI alone in tumour staging local excisions Table S3 Clinical versus pathological tumour staging by MRI + ERUS in local excisions Table S4 Accuracy of MRI + ERUS in tumour staging local excisions [file BJS-107-1372-s003.docx]

**BJS11590**

**MRI cT1–2 rectal cancer staging accuracy: a population-based study**

R. Detering, S. E. van Oostendorp, V. M. Meyer, S. van Dieren, A. C. R. K. Bos, J. W. T. Dekker, O. Reerink, J. H. T. M. van Waesberghe, C. A. M. Marijnen, L. M. G. Moons, R. G. H. Beets-Tan, R. Hompes, H. L. van Westreenen, P. J. Tanis and J. B. Tuynman, on behalf of the Dutch ColoRectal Cancer Audit Group

**Fig. S1 Study flow chart**


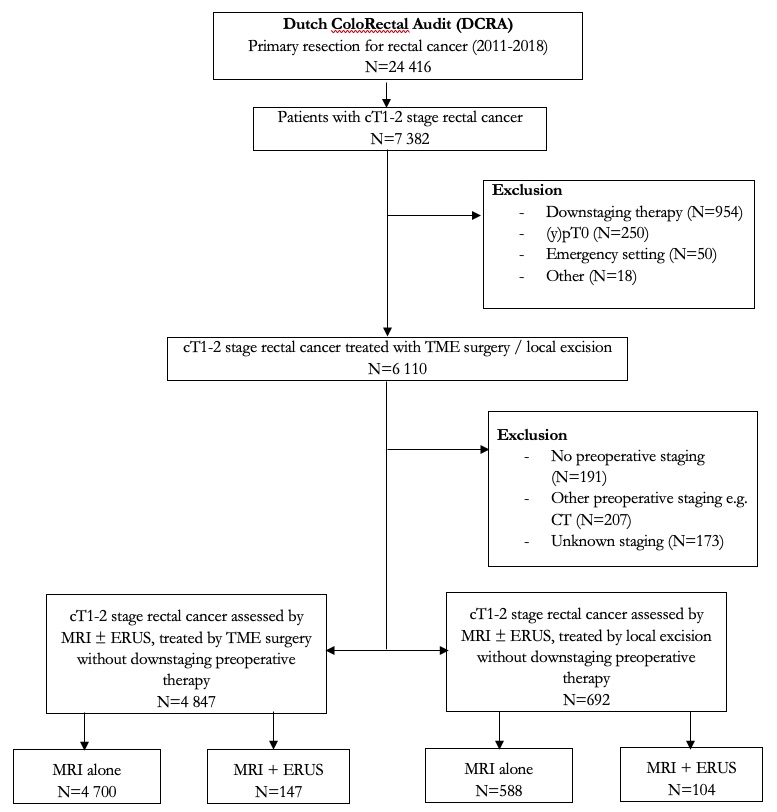


**Fig. S2 Numbers of lymph nodes over time**


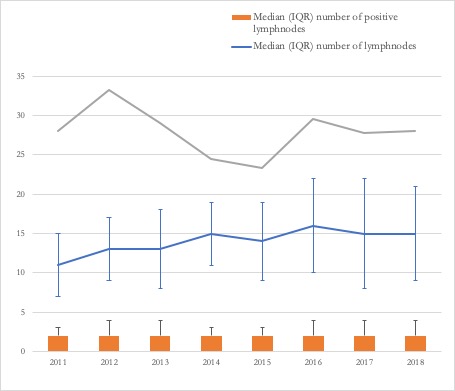


**Table S1 Clinical *versus* pathological tumour staging by MRI alone in local excisions**

| **Clinical *versus* pathological tumour staging** | | | | |
| --- | --- | --- | --- | --- |
|  | **pT1** | **pT2** | **pT3** | **Total** |
| **cT1** | 326 | 66 | 5 | 397 |
| **cT2** | 100 | 80 | 11 | 191 |
| **Total** | 426 | 146 | 16 | 588 |

**Table S2 Accuracy of MRI alone in tumour staging local excisions**

|  | **Accuracy MRI alone tumour staging (% (95% CI))** | | | | |
| --- | --- | --- | --- | --- | --- |
|  | **Sensitivity** | **Specificity** | **PPV** | **NPV** | **Accuracy** |
| cT1 | 76.5 (72.2-80.5) | 56.2 (48.2-64.0) | 82.1 (79.3-84.6) | 47.6 (42.2-53.1) | 70.9 (67.1-74.6) |
| cT2 | 54.8 (46.4-63.0) | 74.9 (70.6-78.9) | 41.9 (36.7-47.3) | 83.4 (80.6-85.8) | 69.9 (66.0-73.6) |

PPV, positive predictive value; NPV, negative predictive value.

**Table S3 Clinical *versus* pathological tumour staging by MRI + ERUS in local**

**excisions**

| **Clinical versus pathological tumour staging** | | | | |
| --- | --- | --- | --- | --- |
|  | **pT1** | **pT2** | **pT3** | **Total** |
| **cT1** | 71 | 13 | 1 | 85 |
| **cT2** | 10 | 9 | 0 | 19 |
| **Total** | 81 | 22 | 1 | 104 |

**Table S4 Accuracy of MRI + ERUS in tumour staging local excisions**

|  | **Accuracy MRI + ERUS tumour staging (% (95% CI))** | | | | |
| --- | --- | --- | --- | --- | --- |
|  | **Sensitivity** | **Specificity** | **PPV** | **NPV** | **Accuracy** |
| cT1 | 87.7 (78.5-93.9) | 39.1 (19.7-61.5) | 83.5 (78.4-87.7) | 47.4 (29.4-66.1) | 76.9 (29.4-66.1) |
| cT2 | 40.9 (20.7-63.6) | 87.8 (78.7-94.0) | 47.4 (29.5-66.0) | 84.7 (79.5-88.8) | 77.9 (68.7-85.4) |

PPV, positive predictive value; NPV, negative predictive value.
